# Hinokiflavone and Related C–O–C-Type Biflavonoids as Anti-cancer Compounds: Properties and Mechanism of Action

**DOI:** 10.1007/s13659-021-00298-w

**Published:** 2021-02-03

**Authors:** Jean-François Goossens, Laurence Goossens, Christian Bailly

**Affiliations:** 1 Univ. Lille, CHU Lille, EA 7365 - GRITA - Groupe de Recherche sur les Formes Injectables et les Technologies Associées, 59000 Lille, France; 2 OncoWitan, 59290 Lille (Wasquehal), France

**Keywords:** Hinokiflavone, Biflavonoid, Cancer therapy, Mechanism of action, Natural product

## Abstract

Biflavonoids are divided in two classes: C–C type compounds represented by the dimeric compound amentoflavone and C–O–C-type compounds typified by hinokiflavone (HNK) with an ether linkage between the two connected apigenin units. This later sub-group of bisflavonyl ethers includes HNK, ochnaflavone, delicaflavone and a few other dimeric compounds, found in a variety of plants, notably *Selaginella* species. A comprehensive review of the anticancer properties and mechanism of action of HNK is provided, to highlight the anti-proliferative and anti-metastatic activities of HNK and derivatives, and HNK-containing plant extracts. The anticancer effects rely on the capacity of HNK to interfere with the ERK1-2/p38/NFκB signaling pathway and the regulation of the expression of the matrix metalloproteinases MMP-2 and MMP-9 (with a potential direct binding to MMP-9). In addition, HNK was found to function as a potent modulator of pre-mRNA splicing, inhibiting the SUMO-specific protease SENP1. As such, HNK represents a rare SENP1 inhibitor of natural origin and a scaffold to design synthetic compounds. Oral formulations of HNK have been elaborated to enhance its solubility, to facilitate the compound delivery and to enhance its anticancer efficacy. The review shed light on the anticancer potential of C–O–C-type biflavonoids and specifically on the pharmacological profile of HNK. This compound deserves further attention as a regulator of pre-mRNA splicing, useful to treat cancers (in particular hepatocellular carcinoma) and other human pathologies.

## Introduction: Biflavonoids

Dimeric flavonoids, usually called biflavonoids, form a specific group of natural products encountered in a large variety of plant species. They are composed of two phenyl-chromenone units, linked via a C–C or C–O–C bond between the chromenone moiety or the appended phenyl ring. The first biflavonoid, ginkgentin was discovered from *Ginkgo biloba* L. in 1929 and, almost one century later, the family includes more than 200 members with a large structural diversity [1].

**Fig. 1:**
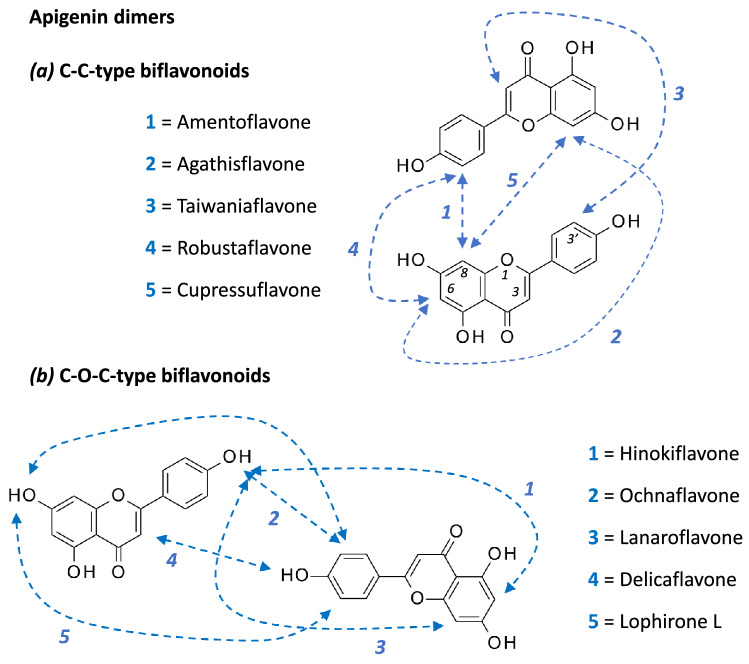
Linkage between two apigenin units to form **a** C–C-type or **b** C–O–C-type biflavonoids

**Fig. 2:**
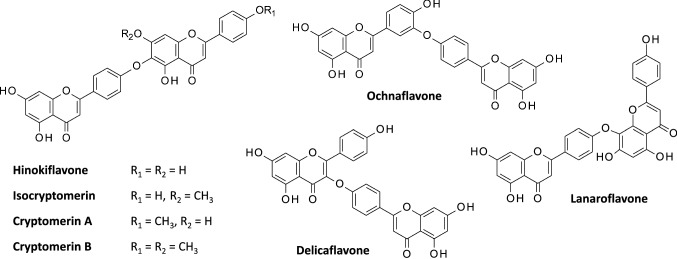
Structures of selected C–O–C-type biflavonoids. They all present an ether linkage between the two apigenin units (or methylated apigenin in some cases)

A motif frequently encountered within biflavonoids corresponds to the dimerization of the apigenin unit (or 4′,5,7-trihydroxy-flavone). There are multiple possible combinations. For example, the linkage of two apigenin motifs via an 8 → 3′ connector affords amentoflavone whereas the connection via an 8 → 6 linker affords agathisflavone. Other combinations are represented in Fig. 1a. The dimerization can also occur via an ether linkage, using one of the hydroxyl groups of apigenin, as shown in Fig. 1b. This is the case for compounds like hinokiflavone, ochnaflavone and a few other C–O–C-type biflavonoids (Fig. 2).

Hinokiflavone (HNK) is a bis-apigenyl ether (Fig. 2) discovered in 1958 in Japan. It was first isolated from the dried leaves of the plant *Chamaecyparis obtusa* Endlicher (also known as Hinoki cypress, Japanese false cypress) [2, 3]. Its structure was fully elucidated one year later [4]. Over the past sixty years, the compound has been isolated from numerous plants (primarily in gymnosperms), such as *Toxicodendron succedaneum, Isophysis tasmanica*, *Juniperus rigida, J. phoenicea, Platycladi cacumen*, *Rhus succedanea*, *Selaginella tamariscina, S. bryopteris*, *Metasequoia glyptostroboides* and many other plants [5–11]. It can be obtained also by total synthesis [12–15]. This apigenin dimer presents an extended V-shaped configuration, similar to that of ochnaflavone, also a C–O–C-type biflavonoid composed of an apigenin and a luteolin subunit (Fig. 2).

Biflavonoids display a large range of biological properties. Some compounds present marked antiviral activities, such as robustaflavone which potently inhibits hepatitis B virus replication [16]. Other compounds display antibacterial properties, like amentoflavone which efficiently kills cyanobacteria [17]. Some compounds also present marked antiproliferative activity, like agasthisflavone [18]. These three natural products–robustaflavone, amentoflavone and agasthisflavone–are C–C-type biflavonoids, which have been previously reviewed [19, 20]. Here, we mainly focused on C–O–C-type biflavonoids and in particular the leading compound in the series, HNK. A review of the antitumor activity and mechanism of action of HNK is offered.

## Pharmacological Profile of HNK

**Fig. 3:**
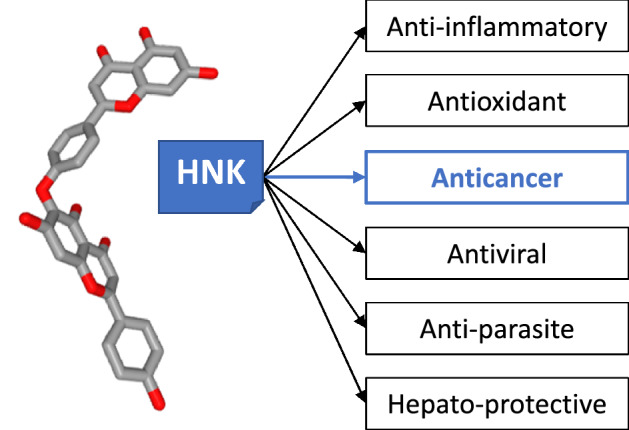
Tridimensional representation of hinokiflavone (HNK, C_30_H_18_O_10_, PubChem CID: 5281627) and its diverse pharmacological activities. The anticancer properties are highlighted here

HNK displays multiple pharmacological activities, including anti-inflammatory, antioxidant, antiprotozoal and antitumor activity (Fig. 3). Due to its antioxidant capacity [21], HNK presents a hepato-protective action, enhanced in the presence of glycyrrhizin [22]. In addition, an in silico study has predicted that HNK can bind to and inhibit prostaglandin D2 synthase, thereby being potentially useful to limit hair loss [23] but, as far as we know, the computer prediction has not been validated experimentally. The anti-inflammatory action of biflavonoids is well documented (reviewed in [24]), although there are not many studies of the anti-inflammatory potential with HNK itself. Nevertheless, HNK was found to suppress the production of inflammatory mediators like nitric oxide (NO) and interleukins IL-6 and IL-8 [25]. The combined antioxidant and anti-inflammatory actions have led to the proposal of using biflavonoids for the treatment of Alzheimer’s disease, considering that biflavonoids have a greater capacity to reduce the toxicity of amyloid-β peptide oligomers than the corresponding monoflavonoids [26, 27]. And indeed, amentoflavone is now emerging as a potential regulator of amyloid β40 neurotoxicity in Alzheimer's disease [28–30]. But a recent structure–activity study demonstrated that HNK-type biflavonoids are less effective than the amentoflavonone-type biflavonoids at reducing Aβ40 aggregation [31].

Antiviral effects have been reported. A weak inhibition of HIV reverse transcriptase activity has been described with HNK [32, 33], as well as an activity against influenza virus sialidase, but also very limited [34]. The activity of HNK against different Herpes viruses is modest, with a minimal margin between the active dose and the cytotoxic dose [35]. Nevertheless, it was shown that HNK can inhibit the dengue 2 virus RNA-dependent RNA polymerase (DV-NS5 RdRp), with a submicromolar efficacy, but other biflavonoids such as amentoflavone and robustaflavone are considered more promising inhibitors [36, 37]. Finally, HNK has also revealed antiprotozoal activities, with a marked capacity to inhibit the growth of both parasites *Leishmania donovani* and *Plasmodium falciparum*, at least in vitro (IC_50_ = 2.9 and 2.3 μM, respectively) [8].

## Anticancer Activity of HNK-Containing Plant Extracts

**Fig. 4:**
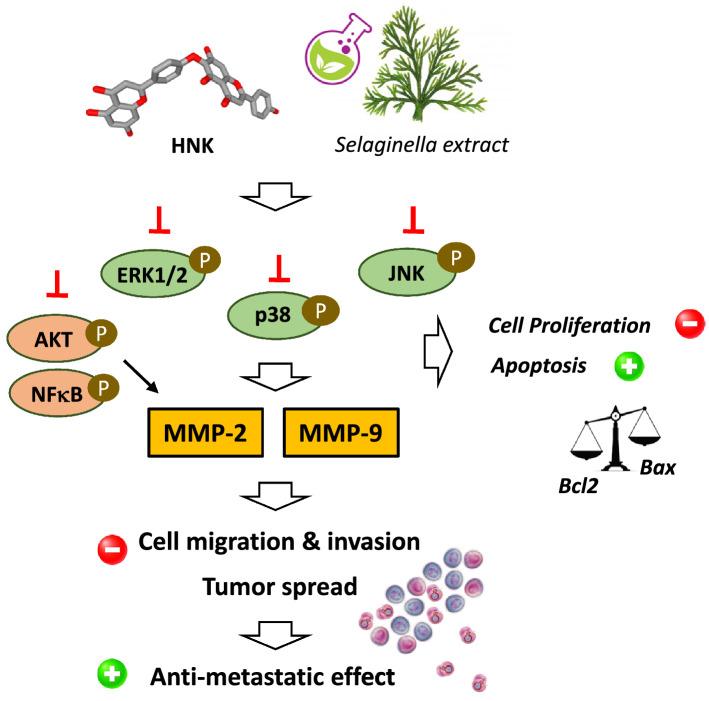
Proposed signaling pathways activated by *Selaginella* extracts and HNK leading to the observed anticancer effect. Down-regulation of matrix metalloproteases MMP-2 and MMP-9 by HNK is a central event largely implicated in the drug-induced reduction of cell migration and invasion, which contributes to inhibition of metastasis

An ethanol extract of the plant *Selaginella tamariscina* used in traditional medicines in Asia was found to display a marked anti-proliferative activity in vitro, against osteosarcoma cell lines. The extract, which contains HNK and other flavonoids, weakly inhibited cell proliferation but markedly reduced cell migration and invasion in a dose-dependent manner. The effect was attributed to a marked down-regulation and inhibition of the matrix metalloproteinases MMP-2 and MMP-9, coupled to an inhibition of the phosphorylation of p38 and Akt signaling molecules [38]. A similar anti-metastatic effect with inhibition of MMP-9 was also reported using human nasopharyngeal carcinoma HONE-1 cells [39]. In general, these biflavonoids including HNK are only mild cytotoxic agents, inhibiting cancer cell growth with IC_50_ in the range 15–40 μM [40]. The anti-metastatic activity of this plant extract has been evidenced in different studies, using leukemia, gastric and lung cancer cells in vitro [41–44] and, also in vivo [45]. The effect has been partially attributed to the presence of amentoflavone, although the extract is known to contain multiple biflavonoids, including HNK but also others, such as pulvinatabiflavone and neocryptomerin [40]. The major characteristic of the extract is to restrict cell migration and invasion (Fig. 4).

Recently, a noticeable anticancer effect was reported using a purified extract of another *Selaginella* specie, namely *Selaginella moellendorffii* Hieron which was found to contain mainly six biflavonoids including HNK [46]. The extract suppressed the migration of laryngeal cancer cells, via an induction of apoptosis and inhibition of STAT3 and the Akt/NFκB signaling pathway. Importantly, the extract showed a dose-dependent activity in vivo, reducing the subcutaneous growth of Hep-2 tumor in mice [46]. HNK is one of the many active compounds that can be found in this type of extract [47]. Both amentoflavone and HNK can be found in various *Selaginella* sp., such as *S. doederleinii* and *S. sinensis* [48–50]. Anticancer effects have been characterized with different types of *Selaginella* extracts, in particular with an ethyl acetate extract of *S. doederleinii* capable of markedly reducing the microvascular density and A549 tumor growth in mice [48, 51].

## Anticancer Properties of HNK

HNK directly affects the proliferation of cancer cells. Early studies showed that HNK inhibited the growth of KB nasopharyngeal cancer cells in vitro, with an ED_50_ of 4 μg/mL, whereas amentoflavone, robustaflavone, and agathisfiavone were inactive against this cell line. However, HNK is a mild cytotoxic agent, being 16-times less potent than the reference drug etoposide against this cell line [52]. Other in vitro studies have reported a modest cytotoxic potential for HNK against various cancer cell lines, with IC_50_ of 19.0, 29.8 and 39.3 μg/mL, against HeLa (cervix), U251 (glioma) and MCF-7 (breast) cancer cells, respectively [40]. Whatever the cell line considered, HNK is almost always more potent than amentoflavone but much less cytotoxic than a conventional cytotoxic drug like cisplatin or etoposide. HNK is active against a variety of cancer cell types, including colorectal cancer cells such as the HT29, HCT116 and CT26 colon cancer cell lines which are roughly equally sensitive to HNK [53]. The cell growth inhibitory action is both time- and drug concentration-dependent, with an inhibitory action on both cell growth and colony formation.

HNK markedly induces apoptosis of colon cancer cells, with an up-regulation of the protein Bax and down-regulation of Bcl-2 and a drug-induced loss of mitochondrial potential. When given daily at 25–50 mg/kg/day, HNK reduced the growth of colon CT26 subcutaneous tumor in mice, without apparent toxicity. The growth inhibitory action in vivo was accompanied with an induction of apoptosis (caspase-3 activation, mitochondrial alterations) and a down-regulation of matrix metalloprotease-9 (MMP-9) [53]. But the effect is not specific to colon cancer cells because much the same activity was reported using A375 and B16 melanoma cells, with an inhibition of cell proliferation, induction of caspase-dependent apoptosis and inhibition of cell migration due to inhibition of MMP-2 and MMP-9 [54]. Inhibition of MMPs is a key element of the mechanism of action of HNK (see below).

The drug-induced modulation of the Bax/Bcl-2 expression ratio (up-regulation of Bax, down-regulation of Bcl-2), associated with a release of cytochrome c and caspases activation has been clearly evidenced using colon [53], hepatocellular [55] and breast [56] cancer cells. In addition, in the case of hepatocellular carcinoma (HCC), HNK was found to inhibit the activation of NFκB (nuclear factor kappa B) signaling, thereby suppressing the expression of several NFκB-target anti-apoptotic genes, and thus reinforcing the direct proapoptotic effect of the compound via up-regulation of phospho-JNK [55]. In the study using a breast cancer model (MDA-MB-231), the authors pointed out the induction of apoptosis as well as a marked anti-metastatic effect. HNK inhibited migration and invasion of breast cancer cells via a modulation of the epithelial‐to‐mesenchymal transition (EMT), specifically through an up‐regulation of the expression level of E‐cadherin and down‐regulation of N‐cadherin [56]. HNK thus displays both antiproliferative and anti-metastatic properties.

It is interesting to compare the level of in vivo activities of HNK in the three independent studies using colon, breast and hepatocellular tumors. In all three cases, the drug significantly slowed down the tumor growth but did not stopp the growth. The effect was relatively modest with the MDA-MB-231 breast cancer model, with a reduction of the tumor volume by 30–40% when HNK was given (intraperitoneal injection) at 20–40 mg/kg [56]. A slightly better effect was obtained when using the CT26 colon cancer model, with a reduction of the tumor volume reaching about 50% when the drug was used at 50 mg/kg [53]. But in the case of HCC, the antitumor effect in vivo was much more pronounced, with a reduction of the volume of SMMC-7721 subcutaneous tumor by 50–70% when the drug was given (ip) at the dose of 4 and 8 mg/kg only. This HCC tumor model seems to be much more sensitive to HNK than the breast and colon cancer models. It should be noted that amentoflavone can also inhibit the growth of HCC tumor cells in mice, but the observed effect was relatively weak, even when the drug was given orally at 100 mg/kg [57]. HNK seems to be much more potent than amentoflavone at inhibiting HCC growth. It would be useful to investigate the combination of HNK with other drugs, such as sorafenib approved to treat advanced HCC.

Whether HNK can also interfere indirectly with cancer cells, via an immune-regulatory action, is not known at present. But this hypothesis is plausible because the related product ochnaflavone presents a T cell immunoregulatory activity, resulting in the production of IL-4 and IL-10 cytokines and suppression of IFN-γ and IL-2 cytokines in a mouse model of fungal (*Candida*) arthritis [58]. Amentoflavone has also been shown to elevate the production of IL-2 and IFN-γ in carcinoma-bearing animals and to enhance natural killer cell activity and lymphocyte proliferation [59]. It would be interesting and timely to determine if HNK also can modulate the immune response and reduce antitumor immunity. The molecular mechanism leading to the anticancer effects of HNK is not precisely known, but at least two categories of protein targets have been evoked. They are discussed below.

## Potential Binding to and Inhibition of Metalloproteases

Matrix metalloproteinases (MMPs) form a class of zinc-dependent peptidase able to remodel the extracellular matrix by favoring tumor invasive processes [60, 61]. Notably, MMP-9 is essential for tumor invasion, metastasis and angiogenesis, and considered as a valid biomarker for cancers [62]. These enzymes are the targets of many natural and synthetic products [63, 64].

Several studies have evidenced the capacity of diverse biflavonoids to inhibit the expression of MMPs, in particular MMP-2 and MMP-9, and this effect is directly implicated in their antitumor action. For example, the C–C type biflavonoids ginkgetin and isoginkgetin both have the capacity to regulate MMPs, reducing the mRNA and protein expression of MMP-2 and MMP-9 and these effects contribute to their anticancer potential [65–67]. Similarly, amentoflavone was found to inhibit metastasis down-regulation of MMP-2 and -9 [68] and to block glioblastoma and osteosarcoma tumor progression through via suppression of the ERK/NFκB signaling pathway, with a down-regulation of MMP-2 and -9 [69, 70]. We can also mention the case of the C–O–C type biflavone ochnaflavone which inhibits MMP-9 secretion in human aortic smooth muscle cells through the transcription factors NFκB and AP-1 [71] or a derivative of agathisflavone which suppresses MMP-2 expression and reduces metastasis of melanoma cells [72]. Other biflavonoids affecting MMPs expressions could be cited [73, 74]. But in general, the biflavonoid-induced down-regulation of MMP-2 and/or -9 is essentially a consequence of an inhibition of the NFκB activity or an upstream signal such as an inhibition of the phosphorylation of extracellular-regulated kinases (pERK-1/2) (Fig. 4). This is the case for HNK which down-regulates the expression of MMP-2 and -9 in A375 and B16 melanoma cells, so as to reduce the invasion/migration capacities of these tumor cells [54]. A down-regulation of MMP-2 was also observed in HNK-treated breast cancer cells [56]. The downregulation of MMPs by HNK can be explained by the modulation of the ERK/NFκB signaling pathway [25].

**Fig. 5:**
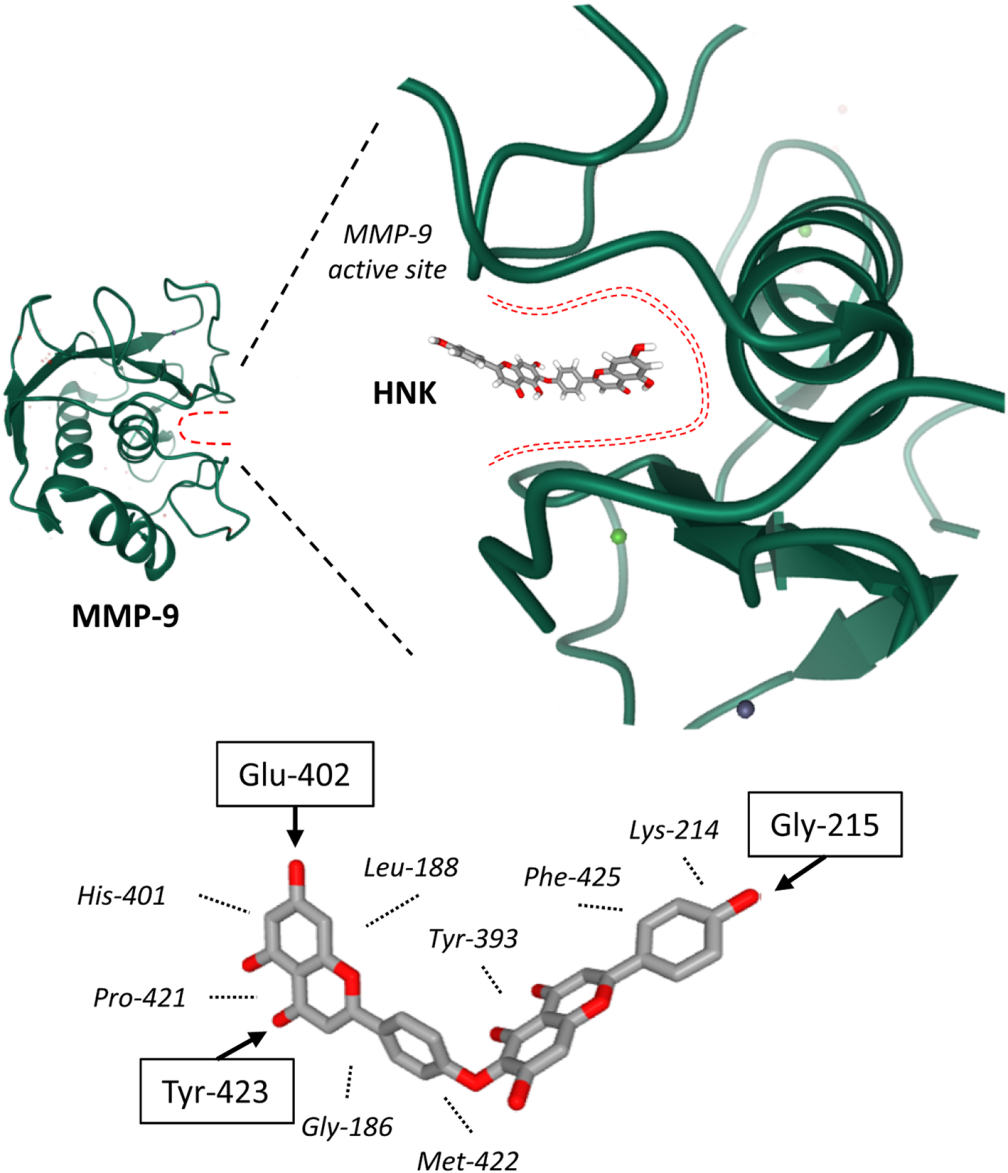
Illustration of the proposed binding of HNK to MMP-9. The structure of MMP-9 is presented (PDB code: 1GKC), with a detailed view of the hinge region which delimits the catalytic active site. Molecular modeling has predicted that HNK can bind deeply into the active site cavity, engaging multiple interactions with the protein, notably through 3 hydrogen bonds with the NH groups of Gly-215, Tyr-423 and C = O group of Glu-402 (arrows), plus hydrophobic contacts with several amino acids (italicized, dashed lines), as represented (adapted from [75])

In parallel, a direct interaction of HNK with MMP-9 has been advanced. A pharmacophore model of MMP-9 has been constructed and potential ligands were screened. HNK turned out to be a suitable binder of MMP-9, forming stable complexes via interaction with the catalytic active site of the protein [75]. In the proposed HNK/MMP-9 model, multiple van der Waals contacts and H-bonds stabilize the biflavone bound to the S1 active site of the protein, as represented in Fig. 5. Each part of the tetracyclic structure of HNK participates in the interaction with MMP-9. Preliminary experimental validation of this in silico hypothesis was provided by the authors who showed that HNK can inhibit MMP-9 activity in cells, with a limited efficacy (IC_50_ = 53 μM) [75]. This is a modest affinity, compared to other known products with submicro-molar affinities [76, 77].

It is worth to note that the formation of a stable biflavonoid/MMP-9 complex has been also proposed with the C–C–type compound amentoflavone [78]. Moreover, MMP-2 and MMP-9 may not be the only metalloproteinases targeted by HNK and other biflavonoids. Recently, it has been proposed, based on an in silico docking study, that lanaroflavone, podocarpusflavone and amentoflavone can bind to the surface metalloprotease leishmanolysin (glycoprotein 63), implicated in the pathogenesis of *Leishmania* [79].

## HNK: A SENP1 Protease Inhibitor Modulating pre-mRNA Splicing

SUMOylation is a post-translational modification whereby members of the Small Ubiquitin-like MOdifier (SUMO) family of proteins are conjugated to lysine residues in target proteins. The SUMOylation process plays a key role in numerous aspects of cell physiology, including cell cycle regulation, protein stability, and DNA-damage repair. SUMO actively regulates transcription, negatively or positively [80]. The deSUMOylation process, carried out by SUMO-specific proteases (SENPs), is equally important and considered as a potential therapeutic target in the treatment of cancers. Small molecules modulators of deSUMOylation are actively searched [81, 82]. Potent SENP-selective inhibitors are now emerging [83] as well as efficient SUMOylation inhibitors, like the anticancer trihydroxyflavone derivative 2-D08 [84–86]. A deregulation of the SUMO pathway has been observed in different cancers, such as breast cancer [87] and hepatocellular carcinoma [88].

**Fig. 6:**
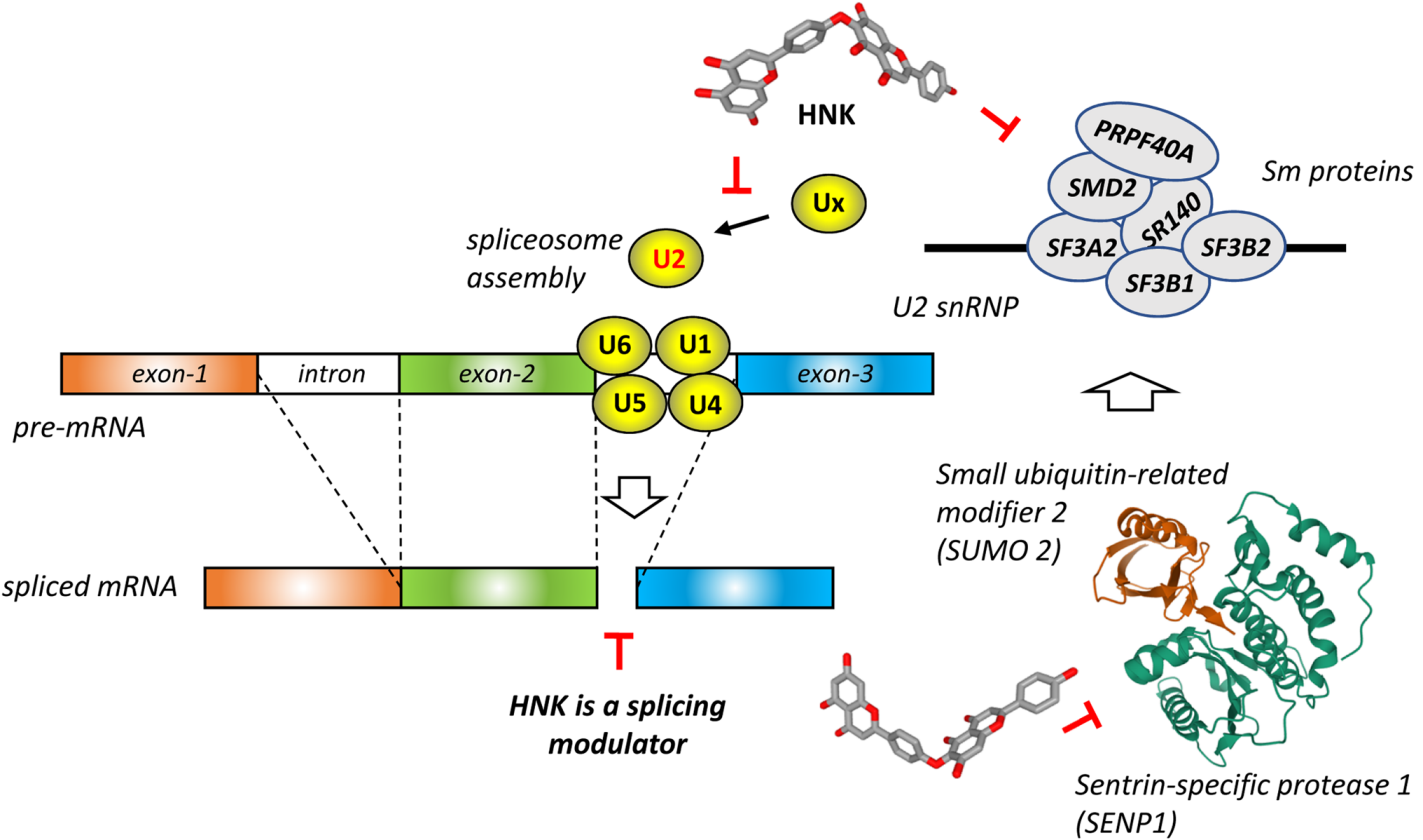
HNK functions as an inhibitor of sentrin-specific protease 1 (SENP1), an essential enzyme for deSUMOylating proteins. The noncovalent structure of SENP1 in complex with SUMO2 is shown (PDB: 6NNQ) [135]. Inhibition of SENP1 by HNK leads to an increased level of six major proteins component (Sm proteins) of the U2 snRNP complex in cells. By this process, HNK induces SUMOylation of splicing factors, thus preventing the correct assembly of the spliceosome and modulating pre-mRNA splicing [89]

Interestingly, Pawellek and co-workers [89] have discovered that HNK functions as an inhibitor of SENP1 in vitro and increased the levels of SUMO2 modification in cells. A treatment with HNK led to a major increase (up to 20-fold) of SUMO1 and SUMO2/3 modification of different proteins in cells, notably a few protein components of the U2 small nuclear ribonucleoprotein (snRNP) spliceosome complex (Fig. 6). The drug was found to block spliceosome assembly and therefore to inhibit mRNA splicing in vitro. The blockade of SENPs by HNK leads to the accumulation of SUMOylated proteins, also observed when using HeLa nuclear extracts [89]. This landmark study provides key information to better comprehend the pharmacological effects of HNK, including its anticancer effects. Indeed, considering that SUMO1 is directly involved in HCC by promoting p65 nuclear translocation and regulating NFκB activity [90], the alteration of the sumoylation/desumoylation machinery by HNK could well be responsible for the observed anticancer effect of the compound in HCC. It is known that SENP1 regulates the migration and epithelial-mesenchymal transition (EMT) of hepatocellular carcinoma [91]. Therefore, inhibition of SNEPs by HNK could explain the observed effects mentioned above, such as the drug-induced modulation of the EMT and reduction of metastasis [56].

Different flavonoids are known to modulate or inhibit mRNA splicing, such as apigenin and luteolin [92–94] and the biflavonoid isoginkgetin was also shown to function as an inhibitor of pre-mRNA splicing [95, 96]. Although this compound is a C–C type biflavonoid structurally distinct from HNK, isoginkgetin appears similar to HNK in terms of mechanism of action, because like HNK, it inhibits tumor cell invasion by regulating MMP-9 expression [65] and interferes with spliceosome assembly, affecting multiple phases of the cell cycle [97]. For example, isoginkgetin efficiently inhibits the splicing of SMN2 (survival of motor neuron 2) mRNA [98]. However, isoginkgetin exerts multiple cellular effects, being also a proteasome inhibitor [99] and a transcription modulator, modifying RNA polymerase elongation rates [100]. In contrast, HNK has no major effect on transcription and is a more potent biflavonoid modulator of splicing than isoginkgetin (and amentoflavone did not alter splicing in vitro) [89].

## Anticancer Activities and Mechanism of Action of HNK Analogues

**Fig. 7:**
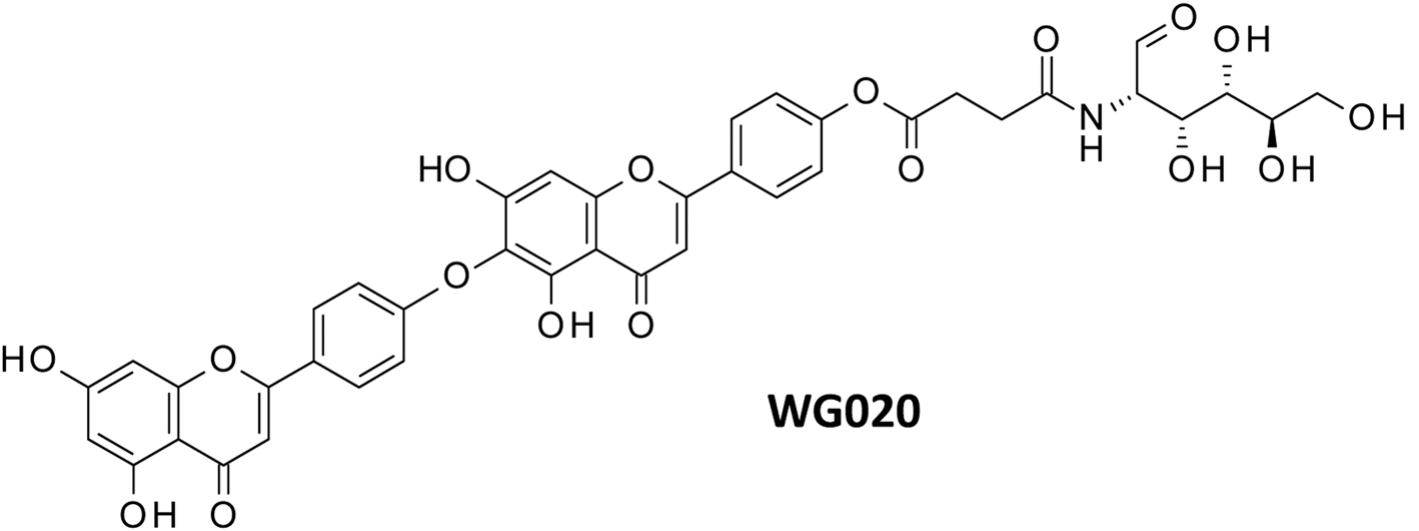
Structure of WG020 [101, 102]

A semisynthetic derivative of HNK has been reported in two Chinese patents recently. The lead compound, designated WG020 (Fig. 7), is a 4′-phenolic ester, prepared by reaction of HNK with succinic acid and amino-glucose. The long polar side chain was incorporated to increase the water solubility and bioavailability of the compound. WG020 displays marked antiproliferative activity, at least in vitro. The compound dose- and time-dependently inhibits the proliferation of breast cancer cells MDA-MB-231, 4T1 and MCF-7. WG020 induces cell apoptosis, characterized by a down-regulation of the anti-apoptotic protein Bcl-2 and up-regulation of pro-apoptotic protein Bax and activation of pro-caspase-3 to complete apoptosis. The compound also inhibits invasion and migration of 4T1 and MDA-MB-231 cells in vitro [101]. Similarly, WG020 inhibits proliferation of human melanoma cells A375 and CHL-1, and murine melanoma cells B16-F10, with IC_50_ in the range 7–11 μM after 72 h and the compound is less toxic toward non-tumoral cells such as VERO and LO2 cells (IC_50_ = 25 μM). WG020 also induces apoptosis of the melanoma cells, again with a marked impact on the expression of Bax and Bcl-2 and with a marked drug-induced decrease of the mitochondrial membrane potential and variations of the production of reactive oxygen species. WG020 reduces migration and invasion of A375 cells [102]. This compound represents a developable form of HNK. After administration, the pro-drug WG020 will be cleaved by internal esterases to release the HNK active unit. Its pharmacokinetic properties have not yet been reported. A prenyl analogue of HNK, made by enzymatic linkage of a geranyl group to the 3’’ position, has been described recently [103] but its bioactivity has not been presented.

With more than 200 biflavonoids identified to date, it is not possible to summarize the biological properties of all compounds but, to complete the review, it is useful to refer to the anticancer properties of selected HNK-like compounds with a C–O–C linkage. Lanaroflavone B is an anti-inflammatory biflavonoid and the formation of stable complexes with human neutrophil elastase has been proposed, based on molecular modeling [104], in addition to the aforementioned binding to leishmanolysin [79]. Ochnaflavone is also an anti-inflammatory agent with a dual cyclooxygenase-2/5-lipoxygenase inhibitory activity [105] but it can also inhibit other enzymes such as phospholipase A2 [106] and suppresses lymphocyte proliferation [107]. But in the context of our review, the most interesting HNK analog is delicaflavone (Fig. 2), isolated from *Selaginella doederleinii*, which has revealed marked anticancer activities in vitro and in vivo, via an inhibition of PI3K/AKT/mTOR and MAPK signaling cascade [108–110]. However, its molecular targets have not been identified yet.

Other biflavonoids have revealed interesting anticancer properties recently, such as robustaflavone [111], isoginkgetin [99], japoflavone D [112], sumaflavone [113] and cupressuflavone [113, 114], but these C–C-type biflavonoids will not be further discussed here.

## Stability, Metabolism and Formulation of HNK

The metabolism of HNK has been investigated recently [115]. More than 40 metabolites have been identified in vitro and in vivo (in rats). The main phase I biotransformation refers to the rupture of the connective C–O–C bond between the two apigenin units, as well and mono- and bi-hydrogenation and hydrolysis of the parent compound. The main phase II metabolism concerns amino acid conjugation (with glutamine, glycine or cysteine), acetylation, and glucuronidation reactions. The metabolites identified are extremely diversified (49 and 41 metabolites identified in vitro and in vivo, respectively), including 25 metabolites only observed in vitro and 24 metabolites found both in vitro and in vivo [115]. Thus, the natural product is largely metabolized but, nevertheless, the elimination of the natural product is not excessively rapid. A pharmacokinetic study in rat indicated that the half-life of HNK elimination (*t*_1/2_) was 6.1 h and the area under the plasma concentration–time curve value (AUC_0-∞_) was about 2500 h × ng/mL [116].

**Fig. 8:**
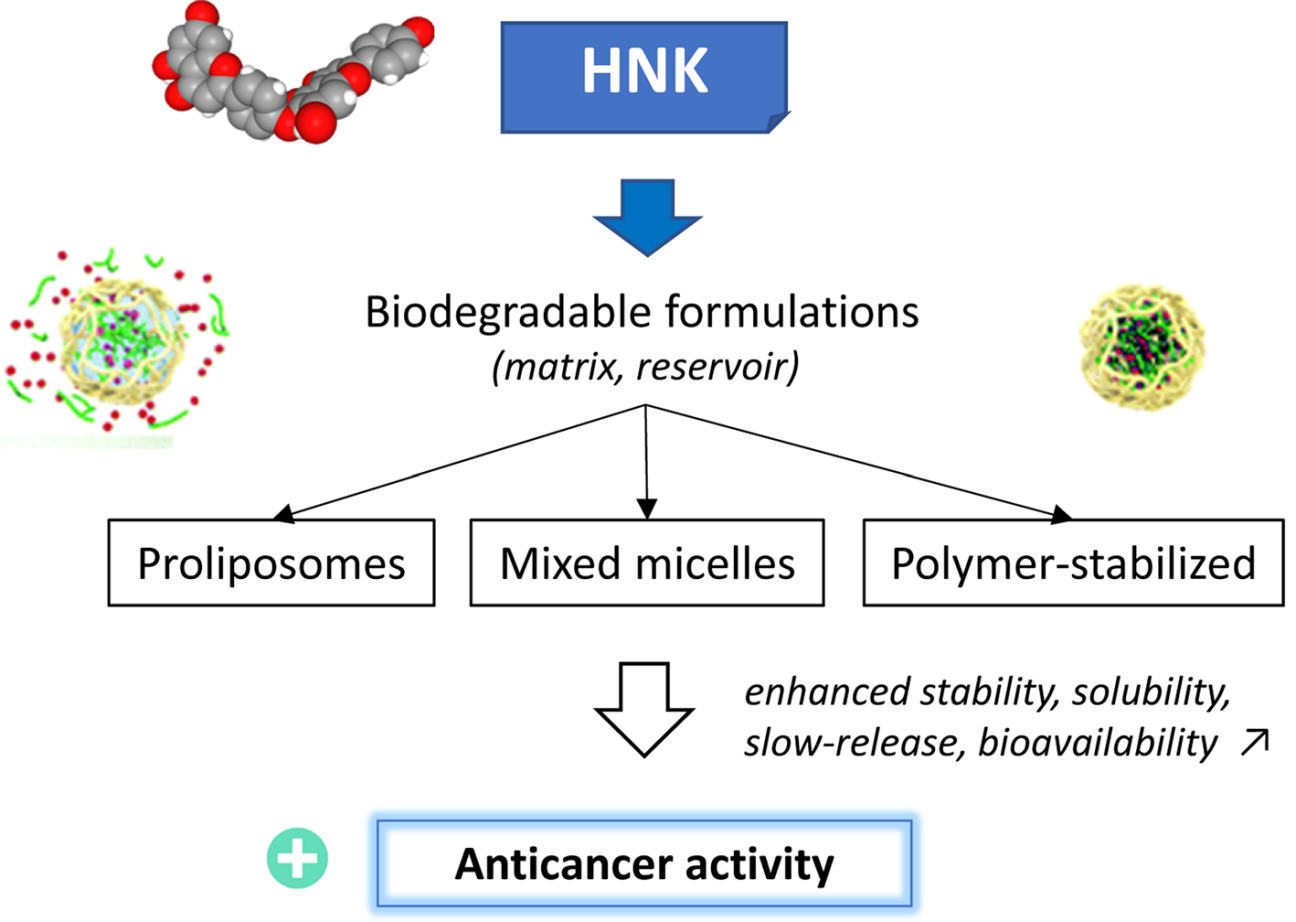
Stable formulations of HNK increase the antitumor effect. Different biodegradable formulations have been proposed to protect the compound, to increase its solubility in aqueous media and its bioavailability. Mixed micelles, proliposomes and amorphous solid dispersion of the polymer-stabilized compounds have been reported. These formulations significantly promote the anticancer activity of HNK or derivatives [107–109]

Oral delivery formulations of HNK have been developed to improve the solubility, dissolution rate, and oral bioavailability of the product (Fig. 8). A mixed micelle formulation of HNK comprising Soluplus®, the lipophilic cation dequalinium, and the nonionic surfactant D-α-tocopherol acid polyethylene glycol 1000 succinate (TPGS, a derivative of vitamin E) has been successfully prepared via a thin-film hydration method, to entrap HNK and to facilitate the drug delivery. The micellar formulation has revealed increased pro-apoptotic and anticancer activities, both in vitro and in vivo compared to the free HNK product. At the dose of 80 mg/kg, the antitumor efficacy of encapsulated HNK was significantly superior to that a free HNK against lung cancer A549-xenografted nude mice [117]. The use of such mixed micelles, characterized by a CMC (critical micelle concentration) value of 5.5 × 10^–4^ mg/mL and an average particle size of 65.6 nm, can be extremely useful to facilitate the oral delivery of the product and enhance its anticancer efficacy, without causing additional toxicity. Other formulations have been developed, such as those applied to promote the efficacy of a biflavonoid extract from *Selaginella doederleinii* (containing both C–C-type and C–O–C-type biflavones) [118, 119]. For example, the use of proliposomes made of a bile salt and a protective hydrophilic isomalto-oligosaccharides coating, has permitted to increase the anticancer efficacy of a biflavonoid extract (containing amentoflavone, robustaflavone, delicaflavone) against a HT29 colon cancer xenograft model [118]. The solubility of the compounds can be vastly improved: for example, the solubility of delicaflavone (C–O–C biflavone) increased from 9.6 μg/mL in water to 188 μg/mL when using a polymer-based formulation prepared by amorphous solid dispersion. The biflavonoid solubility was improved, as well as the dissolution rate of the ingredients and the stability was preserved [119]. These different studies indicate that the encapsulation of HNK or an analog is feasible and recommended to facilitate the handling of the compound and its anticancer efficacy (Fig. 8).

## Discussion

HNK is the leading product of the C–O–C-type sub-group of biflavonoids which also includes lanoroflavone, lophirone L, ochnaflavone and delicaflavone, and a few other compounds such as the methyl esters of HNK, designated cryptomerin A and B [120] and isocryptomerin [121] (Fig. 2). The latter compound is rather an antibacterial and antifungal agent [122, 123] whereas HNK is essentially an anticancer product, like delicaflavone. However, these biflavonoids exhibit a relatively large spectrum of bioactivities, as observed for the C–C-type biflavonoids. Recently, a potential binding of robustaflavone to the protease M^pro^ of SARS-CoV-2 virus has even been postulated [124] and 17 other potential protein targets have been proposed for this compound [20]. Similarly, amentoflavone has revealed multiple targets and pharmacological activities [19], as it is the case with many mono-flavonoids as well. A limited (or obscure) target specificity often restricts the therapeutic applications of these compounds in the clinic [125].

HNK can be found in many plants and contributes to the anticancer effects observed with certain plant extracts, in particular extracts from *Selaginella* sp. It is not a highly cytototoxic agent but a natural product with a good capacity to reduce tumor cell invasion and invasion. It can certainly be easily combined with several conventional anticancer drugs primarily targeting tumor cell proliferation. The anticancer activity of HNK relies, at least in part, on its capacity to interfere with ERK/p38/NFκB signaling pathway and possibly on a direct interaction with MMP-9, but this later aspect remains to be proved experimentally. More importantly, HNK functions as a mRNA splicing modulator and displays a quasi-unique capacity to alter the correct assembly and functioning of the U2 small nuclear ribonucleoprotein (snRNP) spliceosome complex, via an inhibition of SUMO specific protease 1 (SENP1) [89]. Inhibition of SENP1 is very likely the primary effect at the origin of most of the pharmacological properties of HNK. SENP1 is a regulator of key proteins, like the tumor protein suppressor p53. It has been shown recently that SENP1 depletion synergizes with the DNA damage-inducing drug etoposide to induce p53 activation and the expression of p21 [126]. This is exactly the type of effect observed with HNK in hepatocellular carcinoma. HNK was found to inhibit the proliferation of HCC cells via G0/G1 cell cycle arrest with p21/p53 up-regulation [55]. SENP1 is now considered as a valid anticancer target, overexpressed in certain cancer (like non-small cell lung [127] and pancreatic [128] cancers) and a promotor of HCC [129]. Small molecules targeting SENP1 are actively searched [130–132].

Natural products targeting SENP1 are relatively rare but at least two plant products have been reported: the pentacyclic triterpenoid momordin Ic which directly interacts with SENP1 in prostate cancer cells [133] and the triterpenoid triptolide which down-regulates SENP1 in prostate cancer cells [134]. HNK is the third plant product identified as a SENP1 inhibitor, potentially useful for the treatment of cancers, in particular HCC, an essentially incurable inflammation-related cancer for which effective medications are still lacking. The interaction of HNK with SENP1 and the functional consequences of this interaction warrant further investigation, also because SENP1 inhibitors could be useful to treat other human diseases, such as Alzheimer's disease and different CNS pathologies.

In conclusion, the present review highlights the anticancer potential of a category of C–O–C-type biflavonoids, typified by the apigenin dimer hinokiflavone. These bis-apigenyl ethers represent an interesting family of anticancer/antimetastatic agents. Their mechanism of action is likely multi-factorial but importantly, HNK stands as a unique regulator of pre-mRNA splicing, interfering with the SUMO-specific protease SNEP1. Oral formulations of HNK can be elaborated to facilitate the handling of the product and to enhance its anticancer efficacy. This plant natural product provides an original scaffold for the design of novel anticancer drugs targeting SNEP1.

## Abbreviations

HNK: Hinokiflavone
MMP: Matrix metalloproteinase
SUMO: Small ubiquitin-like modifier
